# Pediatric Code Cart Challenge for Emergency Medicine Trainees in Emergency Departments in India

**DOI:** 10.7759/cureus.49722

**Published:** 2023-11-30

**Authors:** Tania Ahluwalia, Serkan Toy, Kaitlyn Boggs, Rachel Hatcliffe, Josh Heffren, Katherine Douglass

**Affiliations:** 1 Pediatric Emergency Medicine, Children's National Medical Center, Washington DC, USA; 2 Emergency Medicine, George Washington University, Washington DC, USA; 3 Departments of Basic Science Education & Health Systems and Implementation Science, Virginia Tech Carilion School of Medicine, Roanoke, USA; 4 Pediatric Emergency Medicine, Medical University of South Carolina, Charleston, USA

**Keywords:** india, emergency departments, trainees, emergency medicine, code cart, pediatric emergency medicine

## Abstract

Background: Code carts provide accessible emergency medication, supplies, and equipment to resuscitate a child. Unfortunately, there are limited studies on pediatric code cart use in resource-limited settings, including in India.

Methods: This was a Pediatric Code Cart Challenge for emergency medicine (EM) trainees in India. After receiving education on pediatric code carts, participants created their code carts and submitted a video showcasing their project. Reviewers evaluated each team’s code cart using a rubric. A six-month follow-up survey assessed participants’ use of code carts and their perception and satisfaction.

Results: Forty-nine participants across six sites completed the survey. The median number of pediatric code cart uses in the past six months was two. Materials frequently used from the code cart included medications (76%), followed by airway equipment (59%), and intravenous (IV) equipment (57%). Only 4% of respondents used an intraosseous (IO) catheter. Two of six sites reported modifying their code cart within the past six months by rearranging and/or adding equipment and medications. Local protocols, pediatric advanced life support guidelines, and references from other hospitals led to changes. Most respondents rated the pediatric code cart useful and appreciated its accessibility, ease of use, organization, and equipment. Respondents said they would add more pediatric equipment, including IO supplies, to improve their code cart.

Conclusion: Participating sites now have pediatric medications and equipment accessible and organized in their code carts. Additionally, EM trainees learned what is needed and how to improve their current pediatric code carts. Future steps include expanding this pilot project to additional sites in low- and middle-income countries.

## Introduction

Pediatric code carts provide emergency medication, supplies, and equipment, including but not limited to vascular access, respiratory equipment and monitoring equipment, which are essential to manage acute pediatric resuscitations [1]. Children have unique anatomic, physiologic, and psychosocial needs amplified in emergencies compared to adults [2]. The Ronald Reagan Institute of Emergency Medicine (RRIEM) at George Washington University (GW) has long-standing partnership programs with institutions across India to deliver education and training for emergency medicine (EM) trainees [3,4]. Faculty and trainees anecdotally recognized the need to improve pediatric code carts in emergency departments (EDs).

India has high pediatric mortality, with an under-five mortality rate of 30.6 per 1000 live births, starkly contrasting the rate of 6.2 per 1000 live births in the USA [5-7]. The leading causes of pediatric mortality in India, including diarrheal diseases and neonatal disorders, underscore the importance of pediatric medications, supplies, and equipment as critical components of pediatric readiness [5,8-9]. Maintaining labeled, well-organized, properly stocked, and easily accessible pediatric resources can enhance pediatric readiness, thereby reducing morbidity and mortality [9]. A crucial component of pediatric readiness is the availability of a portable, mobile-stocked pediatric code cart in the ED [10]. Unfortunately, there is limited literature on pediatric code carts and pediatric readiness in resource-limited settings, including India. A systematic review found a need for more literature on the use of code carts for both pediatric and adult patients and the need for code cart modification in resource-limited settings [11]. A study in Ethiopia evaluated the availability of medications and equipment in nine intensive care units and identified notable emergency item shortages, particularly for pediatrics [12]. This pilot project includes a Code Cart Challenge to help EM trainees improve their knowledge and use of pediatric code carts by adapting or developing their own.

## Materials and methods

The study team recruited sites and participants through our partner programs in India affiliated with RRIEM at GW. EM trainees were invited to participate in our Pediatric Code Cart Challenge. The aim was for EM trainees to develop or improve their pediatric-specific code carts. This was determined to be a research study that was exempt from the George Washington University Committee on Human Research, Institutional Review Board under the Department of Health and Human Services regulatory Category 1 (IRB# NCR224388). Participants gave informed consent.

**Figure 1:**
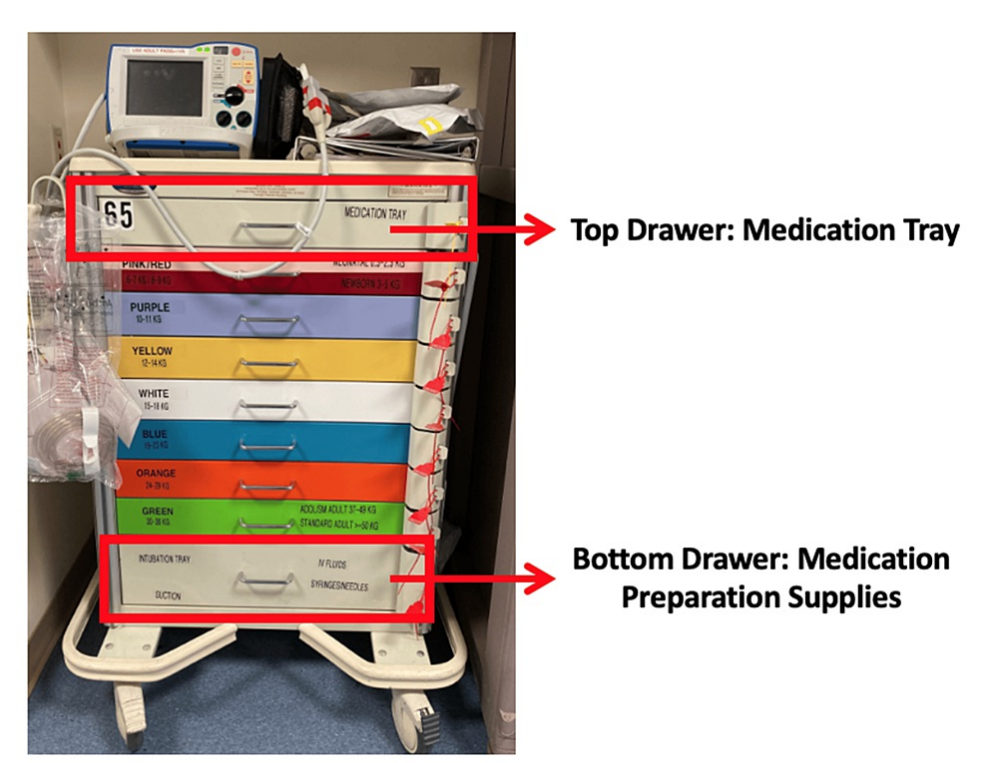
Sample code cart Photo from Children’s National Emergency Department in Washington DC.

**Figure 2:**
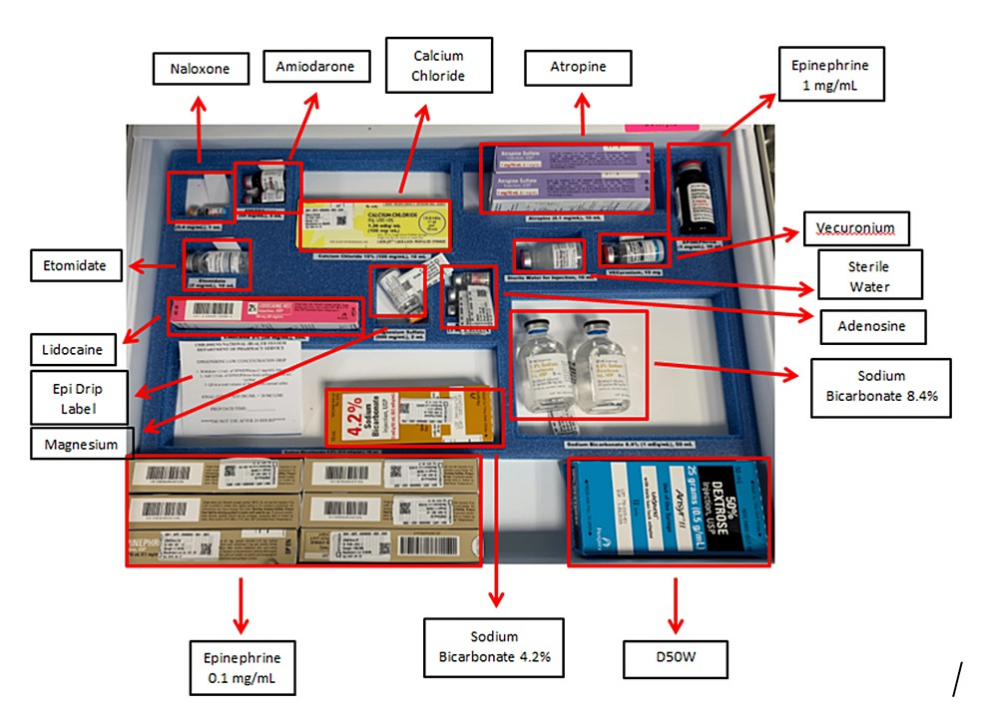
Sample medications From Children’s National Emergency Department in Washington DC.

EM trainees in India were provided education regarding supplies, equipment, and medications for pediatric code carts during didactic sessions via an online platform, Zoom (Zoom Video Communications, Inc., San Jose, CA). Educational content provided an overview and examples of pediatric code carts, medication trays, and reference sheets used at Children’s National Hospital’s Emergency Medicine department in Washington DC, USA (Figures 1, 2). Trainees were encouraged to include local resources in their pediatric code cart. Faculty used email to communicate additional information regarding equipment, supplies, and medications in pediatric code carts.

EM trainees then participated in the Pediatric Code Cart Challenge as a team at each site. The authors developed a rubric to evaluate each code cart and video presentation (Table 1). Participants described their pediatric-specific code cart with a five-minute video submission showcasing their pediatric code cart and explaining how to use it to improve pediatric medical management. Travel restrictions during the coronavirus disease 2019 (COVID-19) pandemic created a significant barrier for US-based faculty to travel to India to assist in person with this project. EM trainees developed pediatric code carts at participating partner programs in India with remote instruction from US-based faculty and guidance from local faculty.

**Table 1:** Evaluation rubric

	0 (lowest)	1	2	3	4 (highest)
Development of a pediatric-specific kit with medications	No medications are included based on available resources	Minimal medications are included based on available resources	Some medications are included based on available resources	Most medications are included based on available resources	All medications included based on available resources
Development of a pediatric-specific kit with equipment (access, airway)	No equipment is included based on available resources	Minimal equipment is included based on available resources	Some equipment is included based on available resources	Most equipment is included based on available resources	All medications included based on available resources
Reference sheet	No reference sheet is included	Minimal information is included on the reference sheet	Some information is included on the reference sheet	Most information is included on the reference sheet	A thorough reference sheet to guide how to use the pediatric-specific kit
Video documentation	No video is provided	Video with minimal information explaining the use of the pediatric-specific kit and reference sheet/guide	Video with some information explaining the use of the pediatric-specific kit and reference sheet/guide and some explanation of how it would improve pediatric resuscitation	Video with most information explaining the use of the pediatric-specific kit and reference sheet/guide and explanation of how it would improve pediatric resuscitation	Detailed video of the pediatric-specific kit and reference sheet/guide and explanation of how it would improve pediatric resuscitation
Global Score (overall effectiveness)	Poor development of a pediatric-specific kit and poor presentation of use	Minimal development of a pediatric-specific kit and sparse presentation of use	Satisfactory development of a pediatric-specific kit and presentation of use	Very satisfactory development of a pediatric-specific kit and presentation of use	Cohesive kit and presentation of use

Key components of the rubric included medications and equipment, as well as a reference sheet based on the pediatric readiness survey [9]. The rubric was reviewed with experts who provided feedback, which led to minor revisions. Video documentation explaining the pediatric code cart and how to use it was included in the scoring. Collectively, this led to a global score for each submission. An evaluation group with key stakeholders, including pediatric EM attendings and fellows, a pediatric resident, an EM resident, and a pediatric EM pharmacist, scored each code cart submission. Each site was provided positive and constructive feedback on their pediatric code cart to improve motivation and engagement through completion.

Participants responded to a six-month follow-up survey to report their use and perception of the pediatric code cart. The survey was developed via REDCap (Research Electronic Data Capture) hosted at GW. The survey had 10 questions and was administered anonymously. Quantitative questions were included to inquire about the participants’ use of the pediatric code carts and frequently used materials within the past six months following the intervention. Participants were also asked if and how they changed or upgraded their pediatric code cart in the past six months and what resources, tools, or experiences they based their changes on. They then rated the code cart on a scale from 0-10, with 10 being the most helpful. Participants were asked to respond to a free-text question to report what they liked most about their pediatric code cart and how it could be improved. Survey results were reviewed, and variables were created, collapsed, and re-categorized. A coding scheme was applied for the written portion of the free text, and two researchers analyzed this data. Survey responses were anonymous.

## Results

Six sites participated in the pediatric code cart challenge (Video 1). Each site collaborated as a team to develop a pediatric code cart at their location, using the education provided as a tool to assist them. Video 1 highlights critical components of the video submissions [13]. The winning submission, Site 1, had a clear pediatric-focused presentation with a code cart based on weight using the Broselow tape. The runner-up, Site 2, showed how they use cognitive aids for pediatric emergencies. They also included different sizes of intraosseous (IO) lines and pediatric cervical collars. Site 3 had reference sheets and used pediatric-specific phone applications. Site 4 included reference books. Site 5 included signs for look-a-like and sound-alike medications. Site 6 had detailed reference sheets with equipment, medications, and quantity.

**Video 1:** Pediatric code cart challenge

Fourty-nine participants completed the survey from five of six sites (Table 2). All participants were in their first, second, or third year of EM training from geographically diverse locations in India. A faculty member disseminated the survey in person. The median number of code cart uses was two, with a minimum of zero uses and a maximum of 10 uses (Table 3). The most frequently used materials from the code cart included medications (76%), followed by airway equipment (59%), and IV equipment (57%).

**Table 2:** Sites that participated in the survey

SITE	N	%
Site 1	12	24.5%
Site 2	8	16.3%
Site 3	7	14.3%
Site 4	18	36.7%
Site 6	4	8.2%
Total	49	100%

**Table 3:** Comparison of sites by their use of code cart in the last six months

SITE	Mean (SD)	N	Median	Min	Max
Site 1	3.17 (2.52)	12	2.00	1	10
Site 2	.87 (1.64)	8	.00	0	4
Site 3	2.43 (1.62)	7	2.00	0	4
Site 4	2.44 (1.25)	18	3.00	1	5
Site 6	.75 (0.96)	4	.50	0	2
Total	2.22 (1.87)	49	2.00	0	10

Only 4% of respondents used IO materials (Table 4). All medications, supplies, and equipment were used for patients, not for simulations or other needs. Two out of six sites (33%) reported modifying their code cart in the last six months. Open-ended responses to how they did this included: “rearranging/changing order”, “getting a new cart”, and “adding equipment and medications”. Resources, tools, or experiences that led to modification include “hospital/local protocol”, “PALS”, “references from other hospitals”, “previous experiences,” and “understanding of lack of required materials while on the case”. 

**Table 4:** Materials used from pediatric code cart IV (intravenous), IO (intraosseous)

Material	Yes n (%)	No n (%)
Airway	29 (59%)	20 (41%)
IV	28 (57%)	21 (43%)
IO	2 (4%)	47 (96%)
Medications	37 (76%)	12 (24%)

**Figure 3:**
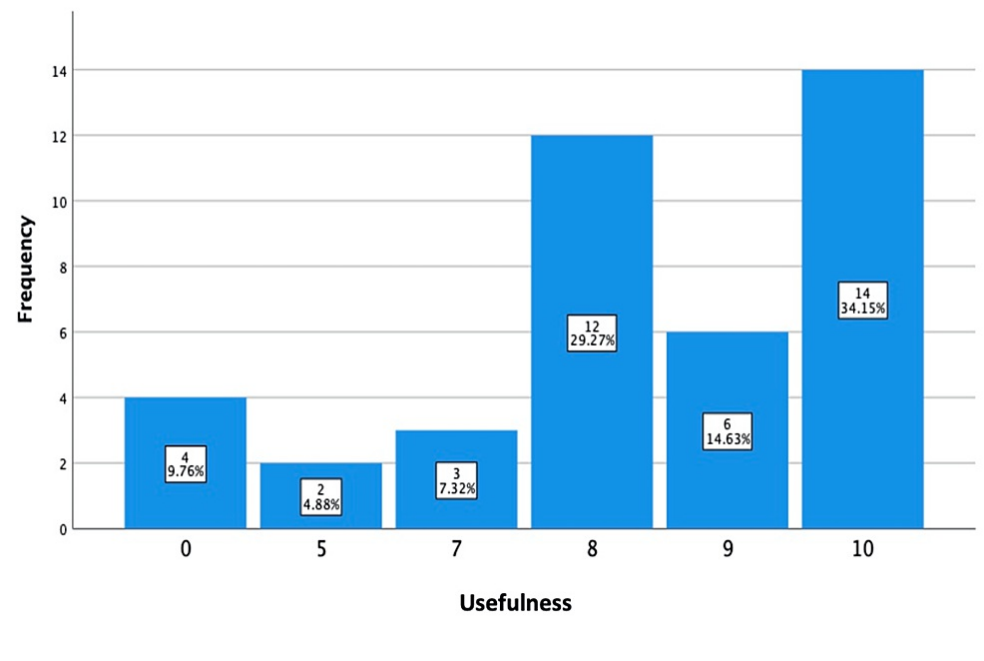
The usefulness of pediatric code carts

Respondents liked their pediatric code cart for its accessibility, described as “ease of access”, “easy to mobilize and handle”, “all medications and pediatric resuscitation equipment are at an arms distance”; dedicated materials in one place; organization described as “the order in which it is arranged”, as well as materials such as “airway equipment”, “IO”, and “Broselow chart”. When asked how their pediatric code cart could be improved, most respondents stated: “include more pediatric equipment,” including “IO cannulation.” Additional suggestions included “ABCD arrangement”, referring to equipment related to airway, breathing, circulation, and disability; “printed protocol posters”, “color coding”, “frequent updating”, and “pediatric drugs with doses”. On a scale from 0-10, with 10 being the most useful, in terms of how useful the pediatric code cart has been during pediatric resuscitations, most respondents (34.15%) rated it 10/10, followed by a rating of 8/10 by 29.27% of respondents (Figure 3).

## Discussion

This pediatric code cart challenge provides practical insights into developing, modifying, and using pediatric code carts in the ED. Six sites participated in the code cart challenge after receiving education. The winning site had a pediatric code cart utilizing a weight-based approach. Additional sites highlighted important elements such as cognitive aids and pediatric-sized equipment such as cervical collars.

Survey results show that respondents found their pediatric code cart useful. While recognizing the need to pay attention to local needs and gaps, participating sites were encouraged to use local equipment and supplies. Interestingly, only 4% of respondents used IOs. There are limited studies that explore the types of materials used for access in the ED in India. One study reviewed anesthesiologist’s use of vascular access for children in India, including arterial, venous (central and peripheral), and intraosseous lines [14]. In resource-limited settings, alternatives to IOs, such as large-bore IV cannulas, may be used to obtain IO access when necessary [15].

This study had several limitations. The small number of participating sites may have yet to provide a representative sample across the country, limiting the potential implications drawn from this study. The COVID-19 pandemic may have played a role in low site participation. Future studies can expand to include additional sites in India as a larger, more diverse population to help improve the generalizability of this study. The reliance on video submissions and participant self-reported surveys may have introduced some bias. As an educational intervention, we chose not to have a control group as we wanted all partner sites to have the option to implement pediatric code carts. However, future studies investigating the effect of pediatric code carts on actual patient outcomes may consider using a control group and more objective measures over time to assess the impact of the intervention.

## Conclusions

In this project, participating sites now have pediatric medications, equipment, and supplies that are more accessible and organized in their code carts. Additionally, EM trainees have gained improved insights into resources for pediatric code carts and how to enhance their existing pediatric code carts. Proficient knowledge and utilization of pediatric code carts, equipped with appropriate pediatric-specific medication, tools, and supplies, are pivotal in improving pediatric readiness in EDs. Future steps for our pilot sites in India involve reviewing resuscitations and long-term outcomes, such as pediatric morbidity and mortality, to guide additional quality improvement initiatives. Furthermore, potential expansion to more locations in India and other low- or middle-income countries is part of our future considerations.
